# Fat redistribution and accumulation of visceral adipose tissue predicts type 2 diabetes risk in middle-aged black South African women: a 13-year longitudinal study

**DOI:** 10.1038/s41387-019-0079-8

**Published:** 2019-03-27

**Authors:** Asanda Mtintsilana, Lisa K. Micklesfield, Elin Chorell, Tommy Olsson, Julia H. Goedecke

**Affiliations:** 1 South African Medical Research Council/University of the Witwatersrand Developmental Pathways for Health Research Unit, Department of Paediatrics, University of Witwatersrand, Johannesburg, South Africa; 2 Department of Public Health and Clinical Medicine, Umeå University, Umeå, Sweden; 3 Non-Communicable Diseases Research Unit, South African Medical Research Council, Cape Town, South Africa

## Abstract

**Background:**

Cross-sectional studies in South Africa (SA) have shown that black SA women, despite being more insulin resistant, have less visceral adipose tissue (VAT) and more subcutaneous adipose tissue (SAT) than white women. This study aimed to investigate whether baseline and/or change in body fat and its distribution predict type 2 diabetes (T2D) risk in middle-aged black SA women, 13 years later.

**Methods:**

We studied 142 black SA women who are the caregivers of the Birth-to-Twenty plus cohort, and who had normal glucose tolerance (NGT) at baseline. At baseline and follow-up, fasting blood samples, basic anthropometry and dual-energy X-ray absorptiometry-derived body composition were measured. At follow-up, an oral glucose tolerance test was completed. The WHO diabetes diagnostic criteria were used to define NGT, impaired fasting glucose (IFG)/impaired glucose tolerance (IGT), impaired glucose metabolism (IGM) and T2D.

**Results:**

At follow-up, 64% of participants remained NGT, whereas 25% developed IGM, and 11% developed T2D. The IGM and the T2D groups were combined for statistical analyses. At baseline, trunk fat mass (FM), VAT but not SAT (measures of central FM) were higher in the IGM/T2D group than the NGT group (*p* < 0.0001). In contrast, the IGM/T2D group had lower leg %FM at baseline than the NGT group (*p* < 0.0001). Baseline trunk FM (Odds ratio per 1 kg increase (95% confidence interval, 1.95 (1.43–2.67))), and VAT (OR per 10 cm^2^ increase, 1.25 (1.10–1.42)), and the change in VAT (1.12 (1.03–1.23)) were associated with greater odds of developing IGM/T2D, whereas baseline leg FM (OR per 1 kg increase, 0.55 (0.41–0.73)) were associated with reduced IGM/T2D risk at follow-up (*p* < 0.05).

**Conclusions:**

Relative fat redistribution, with VAT accumulation, predicted the development of IGM/T2D 13 years before its onset. Prevention of central obesity is a key factor to reduce the risk of developing T2D among middle-aged urban black SA women.

## Introduction

The rapid world-wide increase in obesity levels has led to a rise in the prevalence of non-communicable diseases such as type 2 diabetes (T2D) and cardiovascular diseases, in particular, in low- and middle-income countries such as South Africa (SA)^1,2^. The prevalence of obesity in SA is high, particularly in black women (40.9%)^3^, and accounts for 87% of T2D risk^4^. According to Statistics SA, diabetes mellitus (mainly T2D) was the second cause of death in SA and the leading cause of death among SA women, accounting for 7.2% of all deaths in women in 2016^2^. Notably, the pathophysiology of T2D differs between black and white women^5–9^. Insulin resistance (IR), a major risk factor for T2D, is more pronounced in black SA women compared with their white counterparts even when matched for body fat and waist circumference, and is linked to a greater insulin response to maintain normoglycaemia^5,7–9^. It is the accumulation of central body fat, in particular, visceral adipose tissue (VAT), which mainly determines the risk for IR and T2D^10–12^. Estimation of central body fat via measurement of waist circumference thus does not adequately discriminate between VAT and subcutaneous adipose tissue (SAT)^13^. Interestingly, black SA women are more sensitive to the effects of VAT on hepatic insulin sensitivity than white SA women^8,9^. This could possibly, at least partly, explain the fact that with increasing waist circumference, black women accumulate less VAT, despite being more insulin resistant than their white counterparts^5,8,9^. Notably, these findings are based on cross-sectional studies, mainly conducted in pre-menopausal women^5,8,9^. There is therefore a great need for prospective studies to delineate the possible predictive ability of VAT accumulation, using methods with adequate precision, in the development of T2D among African populations. In particular, studies of middle-aged or older black SA women, who are at high risk of developing T2D, are of main interest in this aspect^14^. This period coincides with menopausal transition in women, and is often characterised by relative redistribution of fat from the peripheral to the central region, and a significant increase in VAT^15–17^. This shift in adipose tissue has been associated with T2D and hypertension risk in pre- and post-menopausal black SA women, respectively^11,18^. There is a lack of studies characterising body fat and fat distribution following menopause in Africans^17–20^ and to our knowledge none have examined whether body fat and fat distribution, including central body fat depots, predict T2D risk in middle-aged or older black SA women. For these reasons, this study in a middle-aged population of urban black SA women aimed to: (i) explore the association between body fat and fat distribution and the risk of developing T2D, 13 years later; and (ii) investigate the independent associations between baseline and/or change in body fat and fat distribution, and measures of glycaemia at 13-year follow-up period.

## Methods

### Study population

**Fig. 1:**
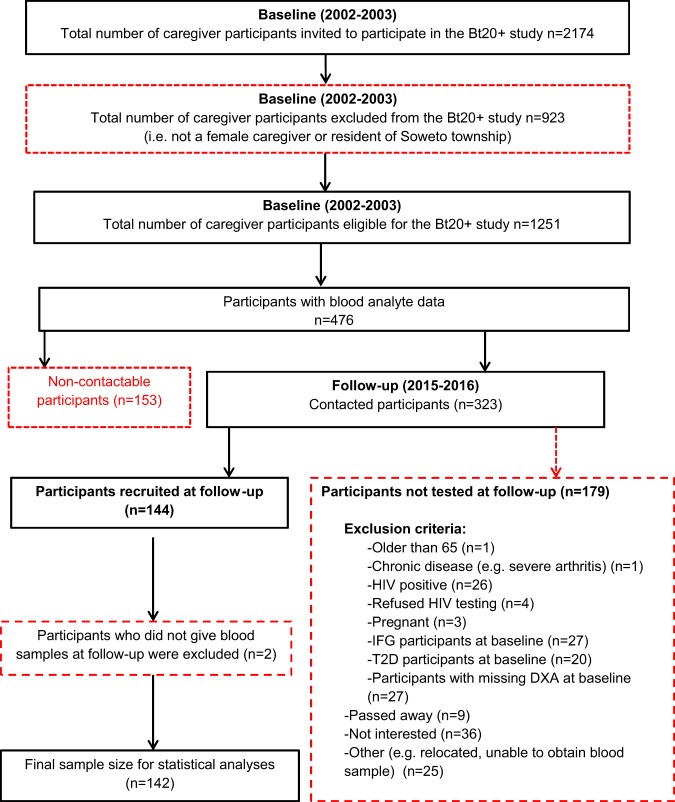
Sample selection flow chart of the female caregivers (e.g., mother, grandmother, aunt) of the Birth-to-Twenty plus (Bt20+) cohort

Baseline data collection was completed between 2002 and 2003 and follow-up took place between 2015 and 2016 (~ 13 years later). In brief, at baseline, 2174 caregivers of the Birth-to-Twenty plus cohort were invited to visit a data collection facility at either Chris Hani Baragwanath Hospital in Soweto or the University of the Witwatersrand Medical School in Johannesburg^21^. However, 1251 were eligible for inclusion in the study and underwent extensive testing that included interviews, whole-body composition assessment by dual-energy X-ray absorptiometry (DXA), and blood sampling^21^ (Fig. 1). Of these participants, few (*n* = 476) had blood analyte data at baseline. At follow-up, contactable participants (*n* = 323) were invited to participate in the follow-up study if they fulfilled the following inclusion criteria: (1) Same female caregiver tested at baseline; (2) < 65 years of age; (3) had a baseline DXA scan; (4) had normal fasting glucose (< 6.1 mmol/l) at baseline; (5) HIV negative on testing. One hundred and seventy nine participants were excluded owing to the strict inclusion criteria and owing to reasons stated in Fig. 1. Of those tested (*n* = 144), blood samples were not obtained from two participants who were then excluded from the statistical analyses. The final sample size for this study was *n* = 142 (Fig. 1).

The study was approved by the Human Research Ethics Committee (Medical) of the University of the Witwatersrand (M010556 and M150530). The procedures and risks associated with the study were explained to the participants and all participants signed the consent form prior to participation in the study. All testing procedures were performed at the South African Medical Research Council/University of the Witwatersrand Developmental Pathways for Health Research Unit at the Chris Hani Baragwanath Hospital in Soweto Johannesburg.

### Body composition

Weight and height, in lightweight clothing without shoes, were measured at baseline and follow-up, using a standard scale and stadiometer, respectively. Waist (level of umbilicus) and hip (largest gluteal area) circumferences were measured in triplicate and the mean used for statistical analyses. Whole-body composition was measured using DXA (Hologic Discovery-W (S/N 71201), Bedford, MA, USA), and included subtotal (whole-body minus head) fat mass and fat-free soft tissue mass. Regional body fat, namely trunk, arm, leg, android, and gynoid fat mass (expressed in kg and as a percentage of subtotal fat mass, % FM) were also measured using DXA cutoff lines positioned at standard anatomical positions, as defined in the software (software version 13.4.2:7). In addition, abdominal VAT and SAT were estimated using algorithms included in the DXA software, which have been shown to perform as well as clinical computed tomography^22^. Baseline and follow-up scans were analysed using the same software (software version 13.4.2:7). Body composition of participants exceeding the scan field limits were calculated using the arm-replacement method^23^. The coefficients of variations during the course of the study were 1 and <2% for fat-free soft tissue mass and total fat mass, respectively.

### Menopausal status

Self-reported menopausal status was determined by questionnaire at follow-up only. Pre-menopause was defined as having a regular menstrual cycle, while peri-menopausal was characterised by menstrual irregularity or having missed 3 months of consecutive menstrual periods. Post-menopause was defined as cessation of the menstrual cycle for > 12 months.

### Blood sampling and biochemical analysis at follow-up

At baseline, fasting blood samples were drawn for the analysis of plasma glucose concentrations^21^. At follow-up, blood samples were drawn in the fasted state (average 12 h) for the subsequent determination of HbA1c (glycated haemoglobin), plasma glucose, and serum insulin concentrations. Thereafter, all non-T2D participants (based on self-reported status and/or use of T2D medication) completed a standard oral glucose tolerance test (OGTT). In brief, participants ingested 75 g of anhydrous glucose dissolved in 250 ml water. Following the glucose ingestion, blood samples were taken at 30, 60, 90, and 120 min. The samples were centrifuged at 3000 rpm for 10 min at 4°C, the plasma was stored at −20°C for subsequent analysis of glucose concentrations, and the serum was stored at −80°C for the analysis of insulin concentrations.

Plasma glucose concentrations were analysed on the Randox RX Daytona Chemistry Analyzer using enzymatic methods (Catalogue number, GL8318) (Randox Laboratories Ltd., London, UK). Serum insulin assays were analysed on the Immulite® 1000 Immunoassay System (Catalogue number, 10381429) (Siemens Chemiluminescent Healthcare GmbH, Henkestr, Germany). HbA1c levels were measured on whole blood samples using the D-10™ Hemoglobin Analyzer (Catalogue number, 2200101) (Bio-Rad Laboratories, Inc., CA, USA).

At baseline, participants were classified as having normal glucose tolerance (NGT) if their fasting glucose data were <6.1 mmol/l^24^. At follow-up, participants were divided into three glycaemic categories using the OGTT results; NGT (fasting glucose < 6.1 mmol/l and 2-h post glucose load < 7.8 mmol/l), impaired fasting glucose (IFG) (fasting glucose ≥ 6.1 and < 7.0 mmol/L and 2-h post glucose load < 7.8 mmol/l, impaired glucose tolerance (IGT) (fasting glucose levels < 7.0 mmol/l and 2-h post glucose load ≥ 7.8 mmol/l and < 11.1 mmol/l) and T2D (fasting glucose ≥ 7.0 mmol/l and/or 2-h post glucose load ≥ 11.1 mmol/l)^24^. Based on these categories, 64.1% participants remained NGT over the 13-year follow-up period, 24.7% and 11.3% progressed to IFG/IGT and T2D, respectively. The IFG/IGT and T2D participants were combined into one group, called the impaired glucose metabolism (IGM) and T2D group (IGM/T2D; *n* = 51) owing to limited sample size and the fact that they had a similar adiposity phenotype; in particular, the IFG/IGT group had a closer body composition profile to the T2D than the NGT group (supplementary Table S1).

IR was calculated from fasting glucose and insulin using the Homoeostasis Model Assessment (HOMA2-IR) calculator v2.2.3 (http://www.dtu.ox.ac.uk/homacalculator/)^25^. Fasting and OGTT glucose and insulin data (0, 30, 60, 90, and 120) were used to calculate insulin sensitivity using the Matsuda Index web calculator (http://mmatsuda.diabetes-smc.jp/english.html)^26^. Participants with missing glucose and insulin data from the OGTT (*n* = 13), and diabetic participants (i.e., those already diagnosed post-baseline, *n* = 8) were excluded from these parameters.

### Statistical analysis

Shapiro–Wilk test was used to assess the distribution of continuous variables. Normally distributed data are presented as mean ± standard deviation (SD) while skewed data are presented as median and interquartile range (IQR). Change in subtotal and regional fat mass (expressed as percentage of subtotal FM, % FM) between baseline and follow-up in the whole sample were compared using paired *t* tests, whereas nonparametric variables were compared using the Wilcoxon signed rank test. Differences in body composition between baseline and follow-up were compared between the NGT and IGM/T2D groups, as well as between the three groups (NGT, IFG/IGT, and T2D) using two-way repeated measures analysis of variance (ANOVA). For the ANOVA, skewed data were transformed into normally distributed data and Levene’s test was used to test for equal variance between groups. Logistic regression analysis was performed to determine the odds ratios (ORs) associated with the risk of developing IGM/T2D (outcome variable, *n* = 51) compared with remaining NGT (reference group, *n* = 91) at follow-up. Moreover, multinomial logistic regression analyses were used to determine the relative risk ratios associated with progressing to IFG/IGT and developing T2D, compared with remaining NGT after 13 years. In addition, robust regression analyses were used to assess the independent contribution of baseline or change in body composition, to measures of glycaemia at follow-up, while adjusting for baseline age and fat mass. To determine whether central and peripheral FM variables were independent predictors of T2D risk, DXA-derived measures of central (VAT) and peripheral (leg) FM were included in the robust and logistic regression. Statistical significance was set at *p* < 0.05. All data were analysed using Stata (Version 13.1, Statcorp, College Station, Texas).

## Results

### Changes in body composition over the 13-year follow-up period

Characteristics of the study population at baseline and follow-up are summarised in Table 1. The average follow-up period was 13 (12–14) years and the median (IQR) age of the participants at follow-up was 54 (49–59) years. At follow-up, 27.5% of the participants were pre-menopausal, 9.9% peri-menopausal and 62.7% post-menopausal (data not shown). All measures of body composition increased significantly between baseline and follow-up (*p* < 0.0001). Body weight and BMI increased by an average of 9.3%. Measures of central obesity increased to a greater extent than lower-body FM, with trunk FM increasing on average by 25%, whereas leg FM increased by 12.3%. Within the abdominal region, VAT increased by 41% compared with 16% in SAT.

**Table 1:** Participant characteristics at baseline and follow-up

	Baseline	Follow-up	Absolute change	% Change
Age (years)	42 (37–48)	54 (49–59)	11 (10–12)	27 (23–32)*
*Anthropometry*				
Weight (kg)	77.4 ± 14.7	84.4 ± 17.3	7.0 ± 9.0	9.3 ± 11.6*
BMI (kg/m^2^)	30.9 ± 5.8	33.9 ± 6.8	3.0 ± 3.5	9.3 ± 11.6*
Normal-weight/overweight/obese (%)	20.4/25.4/54.2	8.5/21.1/70.4*		
Waist circumference (cm)	88.2 ± 11.5	99.7 ± 12.7	11.5 ± 7.9	13.4 ± 9.9*
Hip circumference (cm)	114.7 ± 12.8	120.5 ± 13.8	5.9 ± 8.5	5.3 ± 7.1*
Waist-to-hip ratio	0.77 (0.72–0.82)	0.82 (0.79–0.88)	0.05 (0.02–0.09)	6.35 (3.38–11.59)*
*Body composition and body fat distribution*				
Fat-free soft tissue mass (kg)	36.2 (32.1–39.3)	36.5 (33.0–39.8)	0.5 (−1.4–2.3)	1.6 (−3.7–6.9)^#^
Body fat mass (kg)	33.4 ± 10.3	39.2 ± 12.2	5.8 ± 6.3	19.6 ± 22.6*
Body fat (%)	46.7 (42.3 - 50.4)	50.4 (45.9–54.1)	3.4 (1.7–5.7)	
Trunk FM (kg)	15.0 ± 5.2	18.3 ± 6.0	3.2 ± 3.5	25.2 ± 31.1*
Trunk (%FM)	44.6 ± 4.7	46.4 ± 5.6	1.8 ± 3.4	
Arm FM (kg)	3.8 (2.9–4.7)	4.6 (3.5–5.7)	0.8 (0.3–1.3)	26.0 (8.3–37.6)*
Arm (%FM)	11.2 (10.2–12.4)	11.6 (10.6–13.2)	0.5 (−0.1–1.1)	
Leg FM (kg)	14.1 (11.2–17.3)	15.6 (12.1–19.8)	1.8 ± 2.6	12.3 (1.0–25.4)*
Leg (%FM)	44.0 ± 5.5	41.8 ± 6.1	−2.2 ± 3.3	
VAT (cm^2^)	120 (81–163)	162 (122–204)	41 (15–67)	41 (14–72)*
SAT (cm^2^)	426 (285–536)	505 (382 –608)	70 (23–131)	16 (5–36)*

Data presented as means ± SD or median (25th–75th percentiles). *BMI*, body mass index; % FM (expressed as a percentage of subtotal fat mass); *VAT*, visceral adipose tissue area; *SAT*, subcutaneous adipose tissue area. *p < 0.0001 and ^#^*p* < 0.05

### Differences in body composition between NGT and IGM/T2D groups at baseline and follow-up

Differences in body composition between the NGT and IGM/T2D groups at baseline and at follow-up, as well as change between baseline and follow-up, are presented in Table 2. Whereas weight, BMI, hip circumference, fat-free soft tissue mass, body fat mass and % body fat were not different between the two groups at baseline or follow-up, central adiposity measures, namely waist circumference, waist-to-hip ratio, trunk % FM and VAT, as well as arm %FM were significantly higher in the IGM/T2D compared with the NGT group at baseline and follow-up (*p* < 0.05). In contrast, leg % FM was significantly lower in the IGM/T2D group than the NGT group at both time points. We found similar findings when the three glycaemic groups (i.e., NGT, IFG/IGT, and T2D) were assessed independently; both T2D and IFG/IGT groups had higher trunk FM, VAT, and lower leg FM than the NGT group (Supplementary Table S1). Furthermore, the T2D group had higher waist and hip circumference, waist-to-hip ratio, and arm FM than the NGT group, but these differences were not found between the IFG/IGT and NGT groups. When comparing the T2D and IFG/IGT groups, the T2D group had higher waist-to-hip ratio and trunk FM (only significant at baseline) and lower leg FM than the IFG/IGT group (Supplementary Table S1).

**Table 2:** Body composition characteristics between the NGT and IGM/T2D groups at baseline and follow-up

	NGT group (*n* = 91)		IGM/T2D group (*n* = 51)		ANOVA *p* value		
	Baseline	Follow-up	Baseline	Follow-up	Group	Time	Group × time
Age (years)	41 (36–47)	53 (48–59)*	45 (39–49)	56 (51–60)*	0.049	<0.0001	0.160
*Anthropometry*							
Weight (kg)	75.6 (64.5–89.6)	83.0 (70.8–96.4)*	77.6 (69.5–87.5)	79.4 (72.6–100.2)*	0.383	<0.0001	0.363
BMI (kg/m^2^)	30.8 (25.1–35.6)	33.4 (28.6–38.6)*	31.2 (27.3–34.8)	32.6 (28.8–38.1)*	0.459	<0.0001	0.304
Waist circumference (cm)	86.1 ± 10.7	98.0 ± 12.5*	92.0 ± 12.0^aa^	102.7 ± 12.5^a^*	0.007	<0.0001	0.269
Hip circumference (cm)	114.5 (105.6–123.0)	120.2 (110.5–129.0)*	112.5 (106.0–122.0)	116.4 (109.0–128.9)*	0.636	<0.0001	0.874
Waist-to-hip ratio	0.75 (0.70–0.80)	0.82 (0.77–0.85)*	0.81 (0.76–0.86)^aa^	0.85 (0.81–0.90)^aa^*	<0.0001	<0.0001	0.426
*Body composition and body fat distribution*							
Fat-free soft tissue mass (kg)	35.9 (31.4–39.2)	36.7 (32.8–39.8)	36.5 (33.4–40.2)	36.4 (33.4–40.4)	0.099	0.154	0.209
Body fat mass (kg)	31.7 (24.4–40.2)	38.7 (29.5–48.3)*	32.9 (28.1–40.6)	35.7(31.6–47.8)*	0.571	<0.0001	0.280
Body fat (%)	46.7 (40.5–51.1)	51.1 (45.3–54.5) *	46.8 (43.2–49.7)	50.1 (45.9–53.5) *	0.655	<0.0001	0.586
Trunk %FM	43.2 ± 4.3	44.6 ± 5.2*	47.2 ± 4.2^aa^	49.5 ± 4.8^aa^*	<0.0001	<0.0001	0.151
Arm %FM	10.9 (9.9–11.9)	11.5 (10.5–12.5) *	11.7 (10.8–13.1)^aa^	12.3 (10.7–14.0)^a^	0.002	<0.0001	0.172
Leg %FM	45.6 (42.3–49.6)	42.9 (40.2–47.5)*	41.0 (37.3–44.1)^aa^	38.5 (35.4–41.7)^aa^*	<0.0001	<0.0001	0.244
VAT (cm^2^)	102 (71–153)	151 (104 –193)*	136 (103–171)^aa^	174 (150–230)^aa^*	<0.001	<0.0001	0.601
SAT (cm^2^)	410 (274 –539)	520 (376–614)*	445 (322–535)	496 (382–608)*	0.558	<0.0001	0.135

Data presented as means ± SD or median (25th–75th percentiles). The NGT group (*n* = 91) includes participants that remained NGT at follow-up. The IGM/T2D group (*n* = 51) consist of participants that developed IFG/IGT or T2D over the 13-year follow-up period. *BMI*, body mass index; % FM (expressed as a percentage of subtotal fat mass); *VAT*, visceral adipose tissue area; *SAT*, subcutaneous adipose tissue area.

^a^*p* < 0.05, ^aa^*p* < 0.01 IGM/T2D group vs NGT group at baseline and/or follow-up

*p < 0.0001 Baseline vs follow-up within a defined group

In both groups (i.e., NGT and IGM/T2D), all anthropometric measurements, subtotal FM and DXA-derived measures of central FM (trunk % FM, VAT and SAT) and arm % FM increased significantly between baseline and follow-up, (all *p* < 0.0001), whereas lower-body peripheral adiposity (leg % FM) decreased over time (all *p* < 0.0001). Notably, menopausal status did not differ between groups with 39.8 vs 21.6% of the NGT vs IGM/T2D groups being self-reported pre-menopausal, 9.9% vs 9.8 peri-menopausal and 59.3 vs 68.6% post-menopausal, respectively (*p* = 0.503).

### DXA-derived body composition measures as predictors of IGM/T2D risk at follow-up

The ORs associated with developing IGM/T2D compared with remaining NGT at follow-up, determined by baseline and change in DXA-derived body composition measures, adjusted for the potential effects of age and body fat mass at baseline, are presented in Table 3. The baseline and change in body fat mass were not significantly associated with the risk of IGM/T2D at follow-up. In contrast, greater trunk FM at baseline was significantly associated with approximately twofold greater risk (OR: 1.92, 95% confidence interval (95% CI): 1.41–2.61) of developing IGM/T2D risk 13 years later, independent of baseline fat mass and age (*p* < 0.05). Furthermore, baseline arm FM (upper body peripheral adiposity) was associated with greater odds of developing IGM/T2D at follow-up (*p* < 0.05). Conversely, greater leg FM at baseline was associated with 45% reduced risk (OR: 0.55, 0.41–0.73) of developing IGM/T2D at follow-up, after adjusting for fat mass and age at baseline (*p* < 0.05). Both baseline and change in VAT were independently associated increased risk (OR: 1.25, 1.10–1.42, and 1.12, 1.03–1.23, respectively) of developing IGM/T2D (*p* < 0.05), whereas there were no associations with SAT. When both VAT and leg FM were included in the same model, baseline VAT showed a tendency for increased IGM/T2D risk, whereas the change in VAT was associated with increased risk of developing IGM/T2D, whereas baseline leg FM was independently associated with decreased IGM/T2D risk (*p* < 0.05).

**Table 3:** Body fat and fat distribution measures as predictors of IGM/T2D risk at follow-up

Variables	Odds ratio	95% CI	*p* value	Model *p* value
*FM (kg)*				
Baseline fat mass (kg)	1.01	0.97–1.04	0.622	0.170
Change fat mass (kg)	0.99	0.93–1.04	0.605	
*Trunk FM (kg)*				
Baseline trunk FM (kg)	1.95	1.43–2.67	<0.0001	<0.0001
Change trunk FM (kg)	1.08	0.97–1.21	0.162	
*Arm FM (kg)*				
Baseline arm FM (kg)	2.73	1.39–5.38	0.004	0.004
Change arm FM (kg)	0.98	0.64–1.51	0.941	
*Leg FM (kg)*				
Baseline leg FM (kg)	0.55	0.41–0.73	<0.0001	<0.0001
Change leg FM (kg)	1.02	0.86–1.21	0.809	
*VAT (10* *cm*^*2*^*)*				
Baseline VAT (10 cm^2^)	1.25	1.10–1.42	<0.0001	<0.001
Change VAT (10 cm^2^)	1.12	1.03–1.23	0.010	
*SAT (10* *cm*^*2*^*)*				
Baseline SAT (10 cm^2^)	1.02	0.96–1.08	0.573	0.201
Change SAT (10 cm^2^)	0.98	0.94–1.03	0.437	
*VAT (10* *cm*^*2*^*) and leg FM (kg)*				
Baseline VAT (10 cm^2^)	1.14	0.98–1.32	0.082	<0.0001
Change VAT (10 cm^2^)	1.13	1.01–1.27	0.037	
Baseline leg FM (kg)	0.65	0.47–0.90	0.008	
Change leg FM (kg)	0.91	0.74–1.11	0.362	

Data are presented as odds ratios, 95% confidence interval (CI), and *p* values adjusted for age. Each model includes baseline body fat and fat distribution measures, the change in the body fat and fat distribution measures as predictor variables. Outcome variables include two groups categorised as either “0”, the NGT group (i.e., NGT participants, reference group (*n* = 91)) or “1”, the IGM/T2D group (i.e., IFG/IGT and T2D participants, the exposure group (*n* = 51)). All the body fat and fat distribution models were adjusted for the potential effects of age at baseline, and regional FM, VAT, and SAT models were also adjusted for baseline body fat mass. *FM*, fat mass; *VAT*, visceral adipose tissue area; *SAT*, subcutaneous adipose tissue area

Similar findings were reported when the multinomial logistic regression was performed to assess the relative risk ratios associated with baseline and change in body fat and fat distribution measures in the progression of NGT to IFG/IGT and T2D, compared with remaining NGT at follow-up (supplementary Table S2).

When menopausal status at follow-up was included as a covariate in the models, baseline DXA-derived measures of central and peripheral adiposity including the change in VAT remained predictors of IGM/T2D risk at follow-up (supplementary Table S3). Menopausal status was not a contributor to IGM/T2D risk at follow-up (supplementary Table S3).

### Associations between DXA-derived body composition measures, and markers of glycaemia at follow-up

The associations reported above were repeated using the continuous measures of glycaemia at follow-up, namely, HbA1c, HOMA2-IR, and Matsuda Index (Table 4). Baseline body fat mass, trunk FM, leg FM, and VAT, but not SAT and arm FM, were associated with HbA1c (only body fat mass and trunk), HOMA2-IR and Matsuda Index at follow-up (*p* < 0.05). In addition, only the change in VAT, and not trunk FM, was associated with HOMA2-IR (positive association) and both measures of central adiposity were negatively associated with Matsuda Index. In contrast, only the change in arm and not leg FM was associated with Matsuda Index at follow-up (*p* < 0.05). Body fat mass accounted for a significant but small (4–7%) proportion of the variance in measures of glycaemia at follow-up, whereas measures of central and peripheral adiposity explained a greater proportion of the variance (8–18 and 9–12%, respectively). When VAT and leg FM were combined in the same model, only baseline and change in VAT were associated with HOMA2-IR and Matsuda Index, accounting for similar variance to the individual models. These associations were independent of menopausal status at follow-up (supplementary Table S4).

**Table 4:** Regression coefficients from robust multiple linear models for the prediction of HbA1c, fasting insulin resistance (HOMA2-IR) and OGTT-derived insulin sensitivity (Matsuda Index) at follow-up

	HbA1c				Insulin resistance (HOMA2-IR)				Insulin sensitivity (Matsuda index)			
	*β*	95% CI	*p* value	*R* ^2^	*β*	95% CI	*p* value	*R* ^2^	*β*	95% CI	*p* value	*R* ^2^
*Body fat mass (kg)*												
Baseline fat mass (kg)	0.01	0.00–0.01	0.008	0.07*	0.03	0.01–0.05	0.002	0.07*	−0.07	−0.12—0.02	0.005	0.04*
Change fat mass (kg)	0.00	−0.01–0.01	0.868		0.02	−0.01–0.05	0.280		−0.06	−0.15–0.02	0.155	
*Trunk FM (kg)*												
Baseline trunk FM (kg)	0.06	0.02–0.10	0.008	0.11*	0.25	0.12–0.38	<0.0001	0.15*	−0.63	−0.97—0.29	<0.0001	0.10*
Change trunk FM (kg)	0.01	−0.01–0.03	0.332		0.05	−0.01–0.10	0.083		−0.15	−0.29—0.01	0.034	
*Arm FM (kg)*												
Baseline arm FM (kg)	−0.02	−0.14–0.09	0.662	0.08	0.10	−0.25–0.44	0.583	0.08*	−0.38	−1.37–0.611	0.448	0.07*
Change arm FM (kg)	0.05	−0.03–0.12	0.203		0.15	−0.08–0.37	0.206		−0.72	−1.37—0.07	0.031	
*Leg FM (kg)*												
Baseline leg FM (kg)	−0.04	−0.08–0.00	0.065	0.09*	−0.19	−0.31—0.07	0.002	0.12*	0.47	0.15–0.78	0.004	0.07*
Change leg FM (kg)	−0.00	−0.03–0.03	0.983		0.04	−0.04–0.12	0.367		−0.10	−0.30–0.10	0.331	
*VAT area (10* *cm*^*2*^*)*												
Baseline VAT (10 cm^2^)	0.02	−0.00–0.03	0.093	0.08*	0.11	0.06–0.16	<0.0001	0.18*	−0.23	−0.38—0.09	0.001	0.11*
Change VAT (10 cm^2^)	0.01	−0.01–0.02	0.454		0.08	0.04–0.12	<0.0001		−0.20	−0.31—0.09	0.001	
*SAT area (10* *cm*^*2*^*)*												
Baseline SAT (10 cm^2^)	0.00	−0.01–0.01	0.385	0.08*	0.01	−0.02–0.05	0.372	0.08*	−0.08	−0.18–0.01	0.093	0.09*
Change SAT (10 cm^2^)	0.00	−0.00–0.01	0.356		0.02	−0.01–0.04	0.186		−0.09	−0.16—0.02	0.013	
*VAT are (10* *cm*^*2*^*) and Leg FM (kg)*												
Baseline VAT (10 cm^2^)	0.01	−0.01–0.03	0.353	0.10*	0.09	0.04–0.15	0.002	0.18*	−0.19	−0.36—0.02	0.027	0.12*
Change VAT (10 cm^2^)	0.00	−0.01–0.02	0.593		0.08	0.03–0.12	0.002		−0.16	−0.22—0.54	0.007	
Baseline leg FM (kg)	−0.03	−0.07–0.02	0.269		−0.07	−0.20–0.06	0.306		0.16	−0.22–0.54	0.409	
Change leg FM (kg)	−0.00	−0.03–0.03	0.784		−0.03	−0.11–0.06	0.556		0.02	−0.21–0.24	0.863	

Data are presented as *β*-coefficients, 95% confidence intervals, *R*^2^ for each model and *p* values. Each model includes baseline body fat and fat distribution measures, the change in the body fat and fat distribution measures as predictor variables and HbA1c, fasting insulin resistance (HOMA2-IR) and OGTT-derived insulin sensitivity (Matsuda Index) measures at follow-up as the outcome variables. All the body fat and fat distribution models were adjusted for the potential effects of age at baseline, and regional FM, VAT and SAT models were also adjusted for baseline body fat mass. *FM*, fat mass. *VAT*, visceral adipose tissue area; *SAT*, subcutaneous adipose tissue area. *P value for the model < 0.05

## Discussion

We show for the first time that both the baseline and change in VAT predicted later development of IGM/T2D in middle-aged black SA women 13 years later. Moreover, only baseline, and not the change, in DXA-derived measures of central (e.g., trunk) and lower-body peripheral (leg) adiposity predicted IGM/T2D at follow-up. Specifically, baseline trunk FM was associated with a twofold increased IGM/T2D risk, whereas leg FM was associated with ±50% reduced risk of developing IGM/T2D.

The finding that both baseline and change in VAT-predicted IGM/T2D risk in black African women is notable as previous studies in SA and the USA have shown that for the same BMI or WC, black women have less VAT and more abdominal and gluteal SAT^5–7,27,28^ than their white counterparts, despite being more insulin resistant^5,7^. Further, a number of studies showed that abdominal SAT, rather than VAT, was the major determinant of IR in black SA and African American women^6,8^. However, all these studies were cross-sectional and included only pre-menopausal women^6,8^. In this first longitudinal study, we show that both baseline VAT and the change in VAT over the 13-year follow-up period, and not SAT, were the major determinants of IGM/T2D. Put into context, we showed that a 10 cm^2^ increase in VAT was associated with a 12% increase risk for IGM/T2D over a period of 13 years, suggesting that the average increase of 41 cm^2^ in VAT in this study population over the 13 years increases the risk of developing IGM/T2D by ~50%. In line with this, we recently showed that obese black women were more sensitive to the effects of VAT on hepatic insulin sensitivity compared with obese white women^9^. Based on these findings, it is clear that VAT, despite lower levels than reported in white women, is also a major determinant of IGM/T2D in black women. Putative underlying mechanisms behind VAT accumulation and development of IR and T2D includes via its hyperlipolytic activity, pro-inflammatory profile, and owing to its anatomical position, higher free-fatty acid influx directly into the portal circulation, resulting in hepatic IR^13,29–31^.

Another important finding was that greater baseline trunk and arm FM and lower leg FM predicted IGM/T2D in black women. Indeed, we consistently showed higher central and lower peripheral adiposity in the IGM/T2D group compared with the NGT group at baseline and follow-up. Similarly, when the three groups were analysed independently, the T2D and IFG/IGT groups had higher trunk FM, VAT and lower leg FM than the NGT group. Whether the three glycaemic groups are analysed independently or the IFG/IGT and T2D combined (i.e., IGM/T2D), the “take-home” message is the same; having high central obesity and low peripheral adiposity at baseline predisposes individuals to the risk of developing T2D later in life.

Although the effects of trunk FM may be mediated via VAT, the protective effects of lower-body peripheral adiposity against IGM/T2D risk may be explained by lower-body SAT acting as a “metabolic sink” that traps excess free fatty acids, thus inducing a favourable metabolic profile^30–32^. Notably, the increase in FM over the 13-year follow-up period was characterised by a relative redistribution of fat from the gluteofemoral region to abdominal region, with a subsequent increase in VAT. This was previously shown in a study including pre-menopausal black SA women, with the centralisation of fat being associated with reduced insulin sensitivity after a 5-year follow-up period^11^. Previously, we have shown downregulation of adipogenic and lipogenic genes in the gluteal depot of obese black SA women^28^, which may explain the relative redistribution of fat from the periphery to the central regions. Importantly, by using DXA we showed that VAT increased to a greater extent than SAT over time, which corroborates previous findings in pre-menopausal women^11^, and may explain why both baseline and change in VAT were associated with reduced insulin sensitivity and predicted IGM/T2D, whereas the protective effects of leg FM were diminished in the presence of VAT. These findings suggest that peripheral and central adiposity are interlinked in the pathophysiology of T2D and that any disruption to their distribution exacerbates the chances of developing T2D.

The findings of our study of middle-aged women contrast to earlier data from pre-menopausal black women, in which VAT was not the most significant predictor of IR^11^. It has been proposed that older women are at greater risk of developing T2D than younger women owing to the effects of menopause^15–17^. The transition from pre-menopause to post-menopause is characterised by a significant decrease in sex hormones, mainly oestrogen^17,19^, which may contribute to an increase in central obesity, in particular VAT^15–17^. We found that menopausal status was a contributor to IR and insulin sensitivity at follow-up (supplementary Table S4). However, even after adjusting for menopausal status at follow-up, VAT was independently associated with markers of glycaemia and T2D risk. These findings suggest that factors other than menopause per se might be responsible for the accumulation of VAT in post-menopausal women. Indeed, several cross-sectional studies showed that VAT was associated with lifestyle factors such as smoking, diet and physical activity^17,33,34^. Furthermore, frequent participation in sports activities was associated with almost 30% reduction in VAT in older (>55 years) as well as in younger (<40 years) women^34^. Therefore, it is possible that a combination of physiological and lifestyle factors might be responsible for the increased central obesity, in particular VAT, over the 13 years, thereby predisposing this population to develop IGM/T2D.

This study has some strengths and weaknesses. Sample size was limited owing to failure to contact and recruit many of the participants at follow-up. The IGM and T2D participants were combined into one group (IGM/T2D) even though the two groups have different phenotypes^24^. Notably, the body composition profile of the IGM group was closer to the body composition profile of the T2D group, suggesting that the IGM group were already at a high risk of developing T2D. We used surrogate measures of hepatic IR (homeostatic model assessment-IR) and peripheral insulin sensitivity (Matsuda Index), instead of the hyperinsulinemic–euglycemic clamp. However, these measures have been shown to be good proxies of IR and insulin sensitivity^35,36^. Furthermore, we did not adjust for lifestyles factors and sex hormones at baseline or the change in these factors over time. Moreover, menopausal status was only collected at follow-up. This study focussed on black SA women (and not men), being a high risk group for development of T2D and its complications^2,3^.

## Conclusion

Greater central fat mass, in particular VAT, and lower leg fat mass predicted IGM/T2D risk in middle-aged black SA women 13 years later. These findings highlight the detrimental effects of fat distribution and VAT accumulation on T2D risk in black middle-aged women. Future studies are required to understand why black women are more sensitive to its effects on insulin sensitivity and T2D risk than white women^9^. Prevention of central obesity is a key factor in reducing the development of T2D among middle-aged urban black SA women.

## Supplementary information

Supporting Tables; S1-S4
