# Supplementary material for: Fat redistribution and accumulation of visceral adipose tissue predicts type 2 diabetes risk in middle-aged black South African women: a 13-year longitudinal study
Source: Nutr Diabetes. 2019 Mar 27;9:12. doi: 10.1038/s41387-019-0079-8 (PMC6437211; doi:10.1038/s41387-019-0079-8)
Supplement: Supplementary file 1 — Supporting Tables; S1-S4 [file 41387_2019_79_MOESM1_ESM.docx]

**Supporting information**

**Supporting Tables**

**Table S1**: Characteristics of the participants according to the three glycaemic groups

**Table S2:** Body fat and fat distribution measures as predictors of IFG/IGT and T2D risk at follow-up

**Table S3:** Body fat and fat distribution measures as predictors of IGM/T2D risk at follow-up, adjusted for menopausal status

**Table S4:** Regression coefficients from robust multiple linear models for the prediction of HbA1c, fasting insulin resistance (HOMA2-IR) and OGTT-derived insulin sensitivity (Matsuda Index) at follow-up, adjusted for menopausal status

**Table S1**: **Characteristics of the participants according to the three glycaemic groups.**

|  | **NGT (n=91)** | | **IFG/IGT (n=35)** | | **T2D (n=16)** | | **ANOVA** | | |
| --- | --- | --- | --- | --- | --- | --- | --- | --- | --- |
|  | **Baseline** | **Follow up** | **Baseline** | **Follow-up** | **Baseline** | **Follow-up** | **Group** | **Time** | **Group x Time** |
| Age (years) | 41 (36-47) | 53 (48-59)^*^ | 46 (38-49) | 57 (50-60)^*^ | 44 (40-46) | 56 (52-58)^*^ | 0.125 | <0.0001 | 0.258 |
| **Anthropometry** | | | | | | | | | |
| Weight (kg) | 75.6 (64.5-89.6) | 83.0 (70.8-96.4)* | 77.1 (67.7-84.9) | 78.6 (72.6-100.2)* | 78.3 (71.0-100.6) | 81.4 (72.3-98.1) | 0.460 | <0.0001 | 0.109 |
| BMI (kg/m^2^) | 30.8 (25.1-35.6) | 33.4 (28.6-38.6)* | 31.2 (26.9-33.7) | 32.6 (29.0-37.4)* | 32. 4 (29.0-37.0) | 32.4 (28.5-38.9) | 0.439 | <0.0001 | 0.072 |
| Waist circumference (cm) | 86.1 ± 10.7 | 98.0 ± 12.5* | 89.1 ± 10.0 | 100.8 ± 10.9* | 98.4 ± 13.9^aa^ | 106.6 ± 14.9*^a^ | 0.003 | <0.0001 | 0.160 |
| Hip circumference (cm) | 114.5 (105.6-123.0) | 120.2 (110.5-129.0)* | 112.0 (104.0-122.0) | 116.2 (110-0-128.0)* | 116.5 (108.0-123.8) | 117.5 (106.0-131.0) | 0.715 | <0.0001 | 0.237 |
| Waist-to-hip ratio | 0.75 (0.70-0.80) | 0.82 (0.77-0.85)* | 0.80 (0.76-0.83)^cc^ | 0.85 (0.80-0.89)*^c^ | 0.86 (0.81-0.88)^aa b^ | 0.88 (0.84-0.91)*^aa b^ | <0.0001 | <0.0001 | 0.715 |
| **Body composition and body fat distribution** | | | | | | | | | |
| Fat-free soft tissue mass (kg) | 36.0 (31.7-39.2) | 36.7 (32.08-39.8) | 36.3 (33.4-39.2) | 35.6 (33.6-39.8) | 38.1 (33.6-43.0) | 37.0 (33.1-42.7) | 0.122 | 0.475 | 0.432 |
| Fat mass (kg) ^#, ##^ | 31.7 (24.4-40.2) | 38.7 (29.5-48.3)* | 32.9 (26.1-38.9) | 35.8 (31.6-47.6)* | 33.7 (29.4-46.6) | 35.0 (29.0-48.2) | 0.743 | <0.0001 | 0.025 |
| Body fat (%) ^#, ##^ | 46.7 (40.5-51.1) | 51.1 (45.3-54.5)* | 46.2 (43.2-49.6) | 50.2 (46.5-53.2)* | 47.7 (43.6-49.4) | 49.1 (44.7-54.0) | 0.896 | <0.0001 | 0.017 |
| Trunk %FM | 43.2 ± 4.3 | 44.6 ± 5.2* | 46.3 ± 3.8^cc^ | 48.6 ± 4.2*^cc^ | 49.0 ± 4.6^aa b^ | 51.3 ± 5.7*^aa^ | <0.0001 | <0.0001 | 0.355 |
| Leg %FM | 45.6 (42.3-49.6) | 42.9 (40.2-47.5)* | 42.6 (38.8-44.2)^cc^ | 39.6 (37.0-42.0)*^cc^ | 39.9 (34.6-41.6)^aa b^ | 36.6 (30.8-38.7)*^aa b^ | <0.0001 | <0.0001 | 0.497 |
| Arm %FM | 10.9 (9.9-11.9) | 11.5 (10.5-12.5)* | 11.4 (10.7-13.0)^c^ | 12.0 (10.2-14.1) | 12.1 (11.2-13.2)^aa^ | 12.6 (11.5-13.8)^a^ | 0.006 | 0.0014 | 0.391 |
| VAT (cm^2^) | 102 (71 -153) | 151 (104 -193)* | 134 (89 -163)^c^ | 173 (147-230)*^c^ | 156 (130-204)^aa^ | 199 (160-249)^*aa^ | 0.0005 | <0.0001 | 0.705 |
| SAT (cm^2^) ^#, ##^ | 410 (274 -539)^a^ | 520 (376-614)* | 445 (286-513) | 478 (381-603)* | 458 (378-600) | 501 (378-620) | 0.663 | <0.0001 | 0.015 |

Data presented as means ± SD or median (25^th^-75^th^ percentiles). The three categorical groups include participants that remained with NGT (n=91), transitioned to IFG/ IGT (, n=35) and those that developed T2D (n=16) at follow-up. BMI, body mass index; %FM (expressed as a percentage of sub-total fat mass); VAT, visceral adipose tissue area; SAT, subcutaneous adipose tissue area.

^a^p<0.05, ^aa^p<0.01 T2D group vs NGT group at baseline and/or follow-up;

^b^p<0.05, ^bb^p<0.01 T2D group vs IFG/IGT group at baseline and/or follow-up;

^c^p<0.05, ^cc^p<0.01 IFG/IGT group vs NGT group at baseline and/or follow-up.

*p<0.05 Baseline vs follow-up within a defined group.

^#^p<0.05 (group-by-time interaction) T2Dvs NGT; ^##^p<0.05 (group-by-time interaction) T2D vs IFG/IGT

**Table S2: Body fat and fat distribution measures as predictors of IFG/IGT and T2D risk at follow-up**

| **Variables** | **Outcome Variables** | **RRR** | **95% CI** | **p-value** | **Model p-value** |
| --- | --- | --- | --- | --- | --- |
| **FM (kg)** | | | | | |
| Baseline fat mass (kg) | NGT to IFG/IGT | 1.00 | 0.96-1.03 | 0.806 | 0.059 |
| Change fat mass (kg) |  | 1.03 | 0.96-1.10 | 0.474 |  |
|  |  |  |  |  |  |
| Baseline fat mass (kg) | NGT to T2D | 1.04 | 0.98-1.09 | 0.212 |  |
| Change fat mass (kg) |  | 0.92 | 0.84-1.00 | 0.047 |  |
| **Trunk FM (kg)** | | | | | |
| Baseline trunk FM (kg) | NGT to IFG/IGT | 1.72 | 1.23-2.41 | **0.002** | <0.0001 |
| Change trunk FM (kg) |  | 1.12 | 0.99-1.28 | 0.078 |  |
|  |  |  |  |  |  |
| Baseline trunk FM (kg) | NGT to T2D | 2.62 | 1.58-4.34 | **<0.0001** |  |
| Change trunk FM (kg) |  | 1.02 | 0.86-1.21 | 0.797 |  |
| **Arm FM (kg)** | | | | | |
| Baseline arm FM (kg) | NGT to IFG/IGT | 2.65 | 1.24-5.67 | **0.012** | 0.009 |
| Change arm FM (kg) |  | 1.16 | 0.71-1.90 | 0.541 |  |
|  |  |  |  |  |  |
| Baseline arm FM (kg) | NGT to T2D | 2.74 | 1.11-6.72 | **0.028** |  |
| Change arm FM (kg) |  | 0.71 | 0.38-1.32 | 0.281 |  |
| **Leg FM (kg)** | | | | | |
| Baseline leg FM (kg) | NGT to IFG/IGT | 0.59 | 0.43-0.80 | **0.001** | <0.0001 |
| Change leg FM (kg) |  | 1.09 | 0.91-1.32 | 0.346 |  |
|  |  |  |  |  |  |
| Baseline arm FM (kg) | NGT to T2D | 0.47 | 0.31-0.73 | **0.001** |  |
| Change arm FM (kg) |  | 0.85 | 0.65-1.12 | 0.254 |  |
| **VAT (10 cm^2^)** | | | | | |
| Baseline VAT (10 cm^2^) | NGT to IFG/IGT | 1.20 | 1.05-1.38 | **0.007** | 0.001 |
| Change VAT (10 cm^2^) |  | 1.11 | 1.01-1.24 | **0.033** |  |
|  |  |  |  |  |  |
| Baseline VAT (10 cm^2^) | NGT to T2D | 1.37 | 1.14-1.64 | **0.001** |  |
| Change VAT (10 cm^2^) |  | 1.16 | 1.03-1.31 | **0.015** |  |
| **SAT (10 cm^2^)** | | | | | |
| Baseline SAT (10 cm^2^) | NGT to IFG/IGT | 1.02 | 0.95-1.08 | 0.582 | 0.073 |
| Change SAT (10 cm^2^) |  | 1.01 | 0.96-1.07 | 0.615 |  |
|  |  |  |  |  |  |
| Baseline SAT (10 cm^2^) | NGT to T2D | 1.02 | 0.93-1.12 | 0.672 |  |
| Change SAT (10 cm^2^) |  | 0.91 | 0.85-0.99 | 0.019 |  |
| **VAT (10 cm^2^) and Leg FM (kg)** | | | | | |
| Baseline VAT (10 cm^2^) | NGT to IFG/IGT | 1.10 | 0.94-1.29 | 0.242 | <0.0001 |
| Change VAT (10 cm^2^) |  | 1.09 | 0.96-1.24 | 0.173 |  |
| Baseline leg FM (kg) |  | 0.66 | 0.46-0.94 | **0.022** |  |
| Change leg FM (kg) |  | 1.01 | 0.81-1.27 | 0.897 |  |
|  |  |  |  |  |  |
| Baseline VAT (10 cm^2^) | NGT to T2D | 1.27 | 1.01-1.59 | **0.041** |  |
| Change VAT (10 cm^2^) |  | 1.25 | 1.05-1.49 | **0.013** |  |
| Baseline leg FM (kg) |  | 0.62 | 0.38-1.00 | **0.048** |  |
| Change leg FM (kg) |  | 0.69 | 0.49-0.96 | **0.026** |  |

Data are presented as relative risk ratios, 95% confidence interval (CI) and p-values adjusted for age. Each model includes baseline body fat and fat distribution measures, the change in the body fat and fat distribution measures as predictor variables. Outcome variables include three groups; NGT (i.e. NGT participants, reference group categorised as “0”), IFG/IGT and T2D (exposure groups; categorised as “1” and “2”). All the body fat and fat distribution models were adjusted for the potential effects of age at baseline, and regional FM, VAT and SAT models were also adjusted for baseline body fat mass. FM, fat mass; VAT, visceral adipose tissue area; RRR, relative risk ratios; SAT, subcutaneous adipose tissue area.

**Table S3: Body fat and fat distribution measures as predictors of IGM/T2D risk at follow-up, adjusted for menopausal status**

| **Variables** | **Odds ratio** | **95% CI** | **p-value** | **Model p-value** |
| --- | --- | --- | --- | --- |
| **FM (kg)** | | | | |
| Baseline fat mass (kg) | 1.01 | 0.97-1.04 | 0.647 | 0.299 |
| Change fat mass (kg) | 0.99 | 0.93-1.04 | 0.613 |  |
| **Menopausal status (follow-up)** | 0.91 | 0.49-1.69 | 0.767 |  |
| **Trunk FM (kg)** | | | | |
| Baseline trunk FM (kg) | 1.92 | 1.41-2.61 | **0.000** | <0.001 |
| Change trunk FM (kg) | 1.08 | 0.97-1.21 | 0.164 |  |
| **Menopausal status (follow-up)** | 0.96 | 0.50-1.86 | 0.915 |  |
| **Arm FM (kg)** | | | | |
| Baseline arm FM (kg) | 2.79 | 1.41-5.52 | **0.003** | 0.007 |
| Change arm FM (kg) | 0.97 | 0.63-1.49 | 0.889 |  |
| **Menopausal status (follow-up)** | 0.95 | 0.51-1.79 | 0.875 |  |
| **Leg FM (kg)** | | | | |
| Baseline leg FM (kg) | 0.56 | 0.42-0.74 | **0.000** | <0.0001 |
| Change leg FM (kg) | 1.01 | 0.86-1.20 | 0.881 |  |
| **Menopausal status (follow-up)** | 1.06 | 0.55-2.07 | 0.855 |  |
| **VAT (10 cm^2^)** | | | | |
| Baseline VAT (10 cm^2^) | 1.26 | 1.11-1.43 | **0.000** | <0.001 |
| Change VAT (10 cm^2^) | 1.14 | 1.04-1.24 | **0.006** |  |
| **Menopausal status (follow-up)** | 0.82 | 0.43-1.56 | 0.547 |  |
| **SAT (10 cm^2^)** | | | | |
| Baseline SAT (10 cm^2^) | 1.02 | 0.96-1.08 | 0.591 | 0.330 |
| Change SAT (10 cm^2^) | 0.98 | 0.94-1.03 | 0.457 |  |
| **Menopausal status (follow-up)** | 0.92 | 0.49-1.71 | 0.786 |  |
| **VAT (10 cm^2^) and Leg FM (kg)** | | | | |
| Baseline VAT (10 cm^2^) | 1.15 | 0.99-1.34 | 0.066 | <0.0001 |
| Change VAT (10 cm^2^) | 1.15 | 1.02-1.30 | **0.021** |  |
| Baseline leg FM (kg) | 0.67 | 0.49-0.92 | **0.013** |  |
| Change leg FM (kg) | 0.89 | 0.73-1.09 | 0.260 |  |
| **Menopausal status (follow-up)** | 0.89 | 0.45-1.77 | 0.746 |  |

Data are presented as odds ratios, 95% confidence interval (CI) and p-values adjusted for age. Each model includes baseline body fat and fat distribution measures, the change in the body fat and fat distribution measures as predictor variables. Outcome variables include two groups; NGT (i.e. NGT participants, reference group) and the IGM/T2D groups (IFG/IGT and T2D participants). All the body fat and fat distribution models were adjusted for the potential effects of age at baseline, and regional FM, VAT and SAT models were also adjusted for baseline body fat mass. FM, fat mass; VAT, visceral adipose tissue area; SAT, subcutaneous adipose tissue area.

**Table S4:** **Regression coefficients from robust multiple linear models for the prediction of HbA1c, fasting insulin resistance (HOMA2-IR) and OGTT-derived insulin sensitivity (Matsuda Index) at follow-up, adjusted for menopausal status**

|  | **HbA1c** | | | | **Insulin resistance (HOMA2-IR)** | | | | **Insulin sensitivity (Matsuda Index)** | | | |
| --- | --- | --- | --- | --- | --- | --- | --- | --- | --- | --- | --- | --- |
|  | **β** | **95% CI** | **p-value** | **R^2^** | **β** | **95% CI** | **p-value** | **R^2^** | **β** | **95% CI** | **p-value** | **R^2^** |
| **Body fat mass (kg)** |  |  |  |  |  |  |  |  |  |  |  |  |
| Baseline fat mass (kg) | 0.01 | 0.00-0.01 | **0.008** | 0.08* | 0.03 | 0.01-0.05 | **0.001** | 0.09* | -0.07 | -0.11—0.02 | **0.008** | 0.06* |
| Change fat mass (kg) | 0.00 | -0.01-0.01 | 0.898 |  | 0.02 | -0.02-0.05 | 0.344 |  | -0.06 | -0.14-0.03 | 0.196 |  |
| **Menopausal status (follow-up)** | 0.07 | -0.04-0.18 | 0.188 |  | 0.29 | -0.04-0.61 | 0.084 |  | -0.09 | -1.73-002 | **0.225** |  |
| **Trunk FM (kg)** |  |  |  |  |  |  |  |  |  |  |  |  |
| Baseline trunk FM (kg) | 0.06 | 0.02-0.11 | **0.004** | 0.12* | 0.26 | 0.12-0.39 | **<0.0001** | 0.17* | -0.63 | -0.96—0.30 | **<0.0001** | 0.12* |
| Change trunk FM (kg) | 0.01 | -0.01-0.03 | 0.336 |  | 0.04 | -0.01-0.10 | 0.105 |  | -0.13 | -0.26-0.01 | 0.063 |  |
| **Menopausal status (follow-up)** | 0.09 | -0.02-0.19 | 0.116 |  | 0.32 | 0.01-0.63 | **0.042** |  | -0.79 | -1.55—0.02 | **0.046** |  |
| **Arm FM (kg)** |  |  |  |  |  |  |  |  |  |  |  |  |
| Baseline arm FM (kg) | -0.02 | -0.13-0.09 | 0.706 | 0.09* | 0.11 | -0.23-0.45 | 0.539 | 0.09* | -0.37 | -1.34-0.60 | 0.456 | 0.09* |
| Change arm FM (kg) | 0.05 | -0.03-0.12 | 0.199 |  | 0.15 | -0.08-0.37 | 0.200 |  | -0.72 | -1.36—0.08 | **0.027** |  |
| **Menopausal status (follow-up)** | 0.07 | -0.04-0.18 | 0.192 |  | 0.30 | -0.03-0.63 | **0.071** |  | -0.96 | -1.86—0.05 | **0.039** |  |
| **Leg FM (kg)** |  |  |  |  |  |  |  |  |  |  |  |  |
| Baseline leg FM (kg) | -0.04 | -0.08—0.00 | **0.045** | 0.10* | -0.20 | -0.32-0.09 | **0.001** | 0.14* | 0.47 | 0.16-0.78 | **0.003** | 0.09* |
| Change leg FM (kg) | -0.00 | -0.03-0.03 | 0.967 |  | 0.04 | -0.04-0.12 | 0.324 |  | -0.10 | -0.30-0.09 | 0.306 |  |
| **Menopausal status (follow-up)** | 0.08 | -0.02-0.19 | 0.121 |  | 0.33 | 0.02-0.64 | **0.040** |  | -0.84 | -1.64—0.04 | **0.040** |  |
| **VAT area** (10 **cm^2^)** |  |  |  |  |  |  |  |  |  |  |  |  |
| Baseline VAT (10 cm^2^) | 0.01 | -0.00-0.03 | **0.105** | 0.09* | 0.10 | 0.05-0.16 | **<0.0001** | 0.19* | -0.23 | -0.37—0.08 | **0.002** | 0.13* |
| Change VAT (10 cm^2^) | 0.01 | -0.01-0.02 | 0.492 |  | 0.07 | 0.03-0.12 | **0.001** |  | -0.19 | -0.31—0.08 | **0.001** |  |
| **Menopausal status (follow-up)** | 0.07 | -0.04-0.17 | 0.224 |  | 0.26 | -0.05-0.56 | 0.095 |  | -0.72 | -1.54-0.10 | 0.086 |  |
| **SAT area (10 cm^2^)** |  |  |  |  |  |  |  |  |  |  |  |  |
| Baseline SAT (10 cm^2^) | 0.00 | -0.01-0.01 | 0.425 | 0.08* | 0.01 | -0.02-0.04 | 0.415 | 0.10 | -0.07 | -0.16-0.03 | 0.164 | 0.07* |
| Change SAT (10 cm^2^) | 0.00 | -0.00-0.01 | 0.423 |  | 0.01 | -0.01-0.04 | 0.294 |  | -0.06 | -0.13-0.01 | 0.069 |  |
| **Menopausal status (follow-up)** | 0.07 | -0.04-0.18 | **0.223** |  | 0.27 | -0.06-0.60 | 0.108 |  | -0.78 | -1.68-0.13 | 0.093 |  |
| **VAT (10 cm^2^) and Leg FM (kg)** |  |  |  |  |  |  |  |  |  |  |  |  |
| Baseline VAT (10 cm^2^) | 0.01 | -0.01-0.03 | 0.403 | 0.10 | 0.09 | 0.03-0.15 | **0.004** | 0.20* | -0.17 | -0.35—0.00 | **0.047** |  |
| Change VAT (10 cm^2^) | 0.00 | -0.01-0.02 | 0.651 |  | 0.07 | 0.03-0.12 | **0.003** |  | -0.16 | -0.29—0.03 | **0.014** |  |
| Baseline leg FM (kg) | -0.03 | -0.08-00.02 | 0.209 |  | -0.09 | -0.22-0.05 | 0.215 |  | 0.20 | -0.19-0.59 | 0.304 |  |
| Change leg FM (kg) | -0.00 | -0.03-0.03 | 0.789 |  | -0.02 | -0.11-0.06 | 0.604 |  | -0.00 | -0.23-0.23 | 0.998 |  |
| **Menopausal status (follow-up)** | 0.08 | -0.03-0.19 | 0.165 |  | 0.28 | -0.02-0.59 | **0.070** |  | -0.75 | -1.57-0.08 | 0.075 |  |

Data are presented as β-coefficients, 95% confidence intervals, R^2^ for each model as well as p-values adjusted for age. Each model includes baseline body fat and fat distribution measures, the change in the body fat and fat distribution measures as predictor variables and HbA1c, fasting insulin resistance (HOMA2-IR) and OGTT-derived insulin sensitivity (Matsuda Index) measures at follow-up as the outcome variables. All the body fat and fat distribution models were adjusted for the potential effects of age at baseline, and regional FM, VAT and SAT models were also adjusted for baseline body fat mass FM, fat mass. VAT, visceral adipose tissue area; SAT, subcutaneous adipose tissue area. *P value for the model <0.05
